# Supplementary material for: MetaRibo-Seq measures translation in microbiomes
Source: Nat Commun. 2020 Jun 29;11:3268. doi: 10.1038/s41467-020-17081-z (PMC7324362; doi:10.1038/s41467-020-17081-z)
Supplement: Supplementary file 10 — Supplementary Data 7 [file 41467_2020_17081_MOESM10_ESM.zip › File2/Confidence_High_Taxonomy/309777_out.krona.html]

Javascript must be enabled to view this page.

members
magnitude
magnitudeUnassigned
count
unassigned
taxon
rank

309777\_out

7

2
7
superkingdom

7
phylum
1239

186801
7
class

186802
order
7

family
1
31979

1
genus
1485

species
1
59620

SRS077502\_contig\_number\_contig-100\_5012.5012

1
family
541000

1
genus
946234

1193534
1
species

SRS049773\_contig\_number\_5626

1392389
genus
5


SRS018049\_contig\_number\_contig-100\_15277.15278SRS043411\_contig\_number\_6120SRS048060\_contig\_number\_contig-100\_20526.20527SRS104636\_contig\_number\_contig-100\_21581.70345SRS1054691\_contig\_number\_12170
1297617
species
5
